# Relationships between soil properties and species establishment in the restoration of mined soils in the Cerrado biome

**DOI:** 10.1371/journal.pone.0277193

**Published:** 2022-11-04

**Authors:** Daniel Alves Vieira, Thalles Oliveira Martins, Renan Augusto Miranda Matias, Pedro Henrique Fernandes Silva, Marco Bruno Xavier Valadão, Pedro Vilela Gondim Barbosa, Alcides Gatto, José Roberto Rodrigues Pinto, Angela Pereira Bussinguer, Fabiana Piontekowski Ribeiro

**Affiliations:** 1 Department of Post-Graduation in Forestry Science, Faculdade de Tecnologia, Universidade de Brasília -UnB, Brasília, DF, Brasília, Brazil; 2 Department of Forest Sciences—School of Agronomy and Food Engineering, Universidade Federal de Goiás–UFG, Goiânia, GO, Brazil; 3 Department of Forest Sciences, Faculdade de Tecnologia, Universidade de Brasília–UnB, Brasília, DF, Brasília, Brazil; Feroze Gandhi Degree College, INDIA

## Abstract

Restoring the ecosystems of the Cerrado biome is challenging considering the diversity of phytophysiognomies present in the biome, some of which are composed of species from different strata (herbaceous, shrubby, and arboreal), which increases the complexity of restructuring the floristic composition. Other factors was involved, such as soil quality, which directly influences the success of restoration, water storage, and nutrients, the financial costs, and a slow ecological process, due to the adverse circumstances found in the area. be restored. The strong anthropogenic interventions by mining processes reduce dramatically the physical and nutritional composition of the soil. We studied two restoration areas in Paracatu, Brazil, to examine their edaphic conditions six years after mining activities ceased and relate them to the status of the restoration process. In 2009, a Cerrado restoration were established in an area previously explored for gravel extraction. Plants were sampled and identified in 11 transects along the planting lines. The diameter base (DB) and total height (HT) were measured. The physical/chemical quality of the soil substrate was determined using a collection of samples in open trenches at four types of points: Cerrado (TC); dead plant pits (TM); seedling pits having living individuals of the most abundant species (TT); and those of the second-most abundant species (TE). *Cecropia pachystachya* Trécul and *Tapirira guianensis* Aubl. were most abundant and demonstrated the potential to thrive in areas degraded by mining having low mortality rates and growth at relatively DB and HT. The physical quality indicators in the gravel pits were not limiting, indicating that substrate preparation was efficient in this regard. The organic matter content in TM, TT, and TE was low in comparison to that of TC, and the chemical conditions in the TE pit substrates were similar to those in TM pits, suggesting *C*. *pachystachya* is a species with good plasticity, whereas *T*. *guianensis* is present in pits with higher levels of phosphorus.

## Introduction

Mining generally does not cover large territorial areas similar to agricultural and livestock activities. This factor is also related to the average size of deforestation related to mining activities, which is typically smaller than 40 ha [1]. Despite this, the negative environmental impacts generated by the activity can be severe [2]. It occurs due to the frequent actions of large excavations for the withdrawal of the resource of interest, which often results in the suppression of native vegetation, removal of the highly fertile surface soil, disruption of natural regeneration, reduction or destruction of habitat, changes in native fauna, and interruption of corridors for gene flow [3].

The exploitation of materials used in construction, such as gravel, causes surface degradation and exposes the altered land to erosion agents. The locations where the original vegetation cover and the topsoil are removed, called borrow areas; have substantially altered biotic regeneration [4, 5]. Thus, restoration initiatives must be more assertive in mining areas.

When restoration occurs under extreme conditions, such as in mining areas, the role of the substrate becomes more important in plant development. Thus, it is important to evaluate the conditions of the substrates in parallel along with the evaluation of plant development. Information about the soil and/or substrate quality must be more complete to elucidate the effectiveness of the restoration procedures applied and their long-term sustainability [6] physical and chemical conditions of the soil being considered indicators of restoration success, and which in many cases are subject to slow recovery of their properties after excessive use [7].

The substrate quality (*e*.*g*., adequate fertility and texture) is essential for species development during the restoration process [8, 9]. In Cerrado biome (*i*.*e*., Brazilian Savanna) species can naturally establish themselves in low-fertility and acid soils [10, 11]. It is common to analyze the chemical properties of the substrate and the ecophysiological characteristics of species separately [12, 13]. Therefore, it is important to analyze the soil, substrate, and species to obtain a complete picture of the process. Our study involved a restoration project implemented after mining activities for gravel extraction. It assessed the development of the seedlings, investigated the physical and chemical properties of the substrate that could be related to plants mortality, and compared the physical and chemical variables of the soil with an area of native Cerrado vegetation.

Thus, the following questions were formulated: a) are seedlings of species native to the Cerrado biome successful in establishing and growing on the mined substrate? b) Is the physical/chemical quality of the substrate in the pits prepared for planting in gravel adequate to allow the development of seedlings? c) Are the limitations of substrate quality more pronounced in the dry or rainy season? Based on these questions, we hypothesized that abundant species of the Cerrado biome could become established under extreme restoration conditions even some substrate is added.

## Material and methods

### Study area

The study area comprised a gravel borrow area located in Paracatu, Minas Gerais (MG), Brazil, at central coordinates 17°13ʹ10.78″S, 46°41ʹ29.79″W, and an altitude of 645 m. The native vegetation near the area is characterized as Cerrado *stricto sensu*, a savannah formation located in the center of Brazil with a high deforestation history. Oxisols are the most type of soil in the Cerrado biome they are naturally low fertility and with high acid.

The planting of native species of the Cerrado biome was conducted in February 2009, with restoration activities only in planting lines (*e*.*g*., subsoiling, revolving the substrate up to 60 cm deep in the line). The pits were 60 cm deep, and each pit was filled with organic fertilizer (2 kg pit^-1^) and 100 g of N-P-K (4-30-16) fertilizer. The spacing used was 3 m × 3 m, and the total area was 4.46 ha.

According to the Köppen climate classification, the regional climate is megathermal (Aw) with dry winters [14]. The climatic periods are well defined: the rainy season is concentrated in the period from October to March (1295 mm), and drought occurs from April to September (136 mm), with an average annual precipitation of 1,431 mm according to the meteorological station in Paracatu, MG between 2010 and 2015 (INMET, 2021). The average annual temperature is 23°C.

### Data collection

In September 2015, *i*.*e*., after 67 months systematic sampling of the seedlings planted in the area during the restoration process was conducted to determine which species were present in greater abundance as well as their dendrometric characteristics. One of the 51 planting lines was initially randomly selected, followed by the selection of the others based on an interval of four lines between the samplings. Consequently, 11 planting lines were sampled, where the number of individuals of planted species (N), total height (HT) (measured with a graduated rod), and diameter base (DB) (measured with a digital caliper) were recorded.

After 6 months was evaluated the mortality of seedlings of *C*. *pachystachya* and *T*. *guianensis*. For this evaluation, the two species with the highest number of individuals in the sample were identified. Then, all individuals were counted in a scan contemplating all planting lines. Individuals of other species were also counted, but only to obtain the general mortality rate of planting. The plants were considered dead when they were in the planting pit, presented dry stems, and were devoid of leaves [15]. The mortality rate was calculated using Eq 1:

$$Mortality=\left(1-\frac{n}{N}\right)*100$$ (1)

where n is the number of living individuals, and N is the number of individuals planted.

To characterize the substrate texture of the gravel and soil under the Cerrado, a particle size analysis of four composite samples, each formed by 20 simple deformed samples, was performed in each location, with the aid of a Dutch Hill tool, in the top 0–20 cm. Determination of substrate quality in the gravel pits and of the soil under the native Cerrado adjacent to the gravel was performed by opening trenches (dimensions 40 × 40 × 40 cm), where substrate/soil samples were collected in the surface (0–20 cm) and subsurface (20–40 cm) layers. Trenches were arranged at four collection points.

TC: Trenches arranged randomly in the remaining area of Cerrado *stricto sensu*, in the vicinity of gravel.TM: Trenches in dead plant pits randomly selected on planting lines.TT: Trenches in the pits of the seedlings of living individuals of the most abundant species based on the sampling of planted seedlings, selected at random in the lines where they occurred. Trenches were opened close to the collection of the plants, and the substrate was collected in the “wall” close to the root system to identify possible physicochemical limitations.

TE: Trenches in the pits of the seedlings of living individuals of the second-most abundant species based on the sampling of the planted seedlings, selected at random in the lines where they occurred, and adopting the same procedure as for TT.

**Fig 1:**
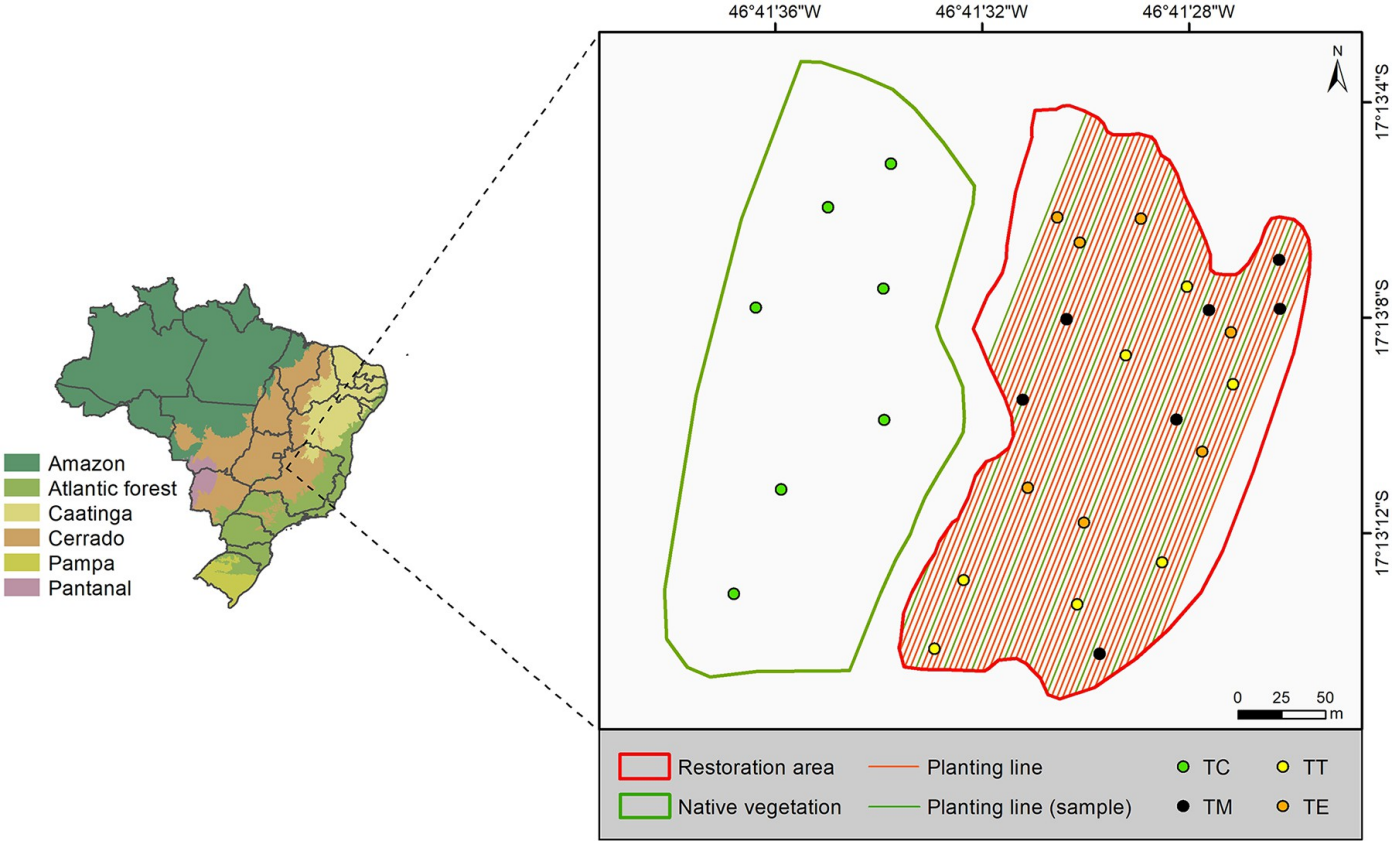
Map of the study area with respective planting lines and sample soil trenches. TC = Samples taken from the soil under Cerrado; TM = Samples taken from pits where seedlings did not survive; TT = Samples taken from *T*. *guianensis* pits; TE = Samples taken from the pits of *C*. *pachystachya*. Source: IBGE [16].

Samples were collected in seven trenches at each described point type (Fig 1). Considering that the dry season occurs from May to September and the rainy season from October to March, the procedures were performed at the end of the dry season (September 2015) and were repeated at the end of the rainy season (March 2016) to determine the extent of limitations in the chemical/physical quality of the substrate in both seasons.

The chemical quality of the substrate of the gravel and soil under the Cerrado was evaluated by the degree of acidity, which included pH (H_2_O), exchangeable aluminum (Al^3+^), potential acidity (H+Al), and aluminum saturation (m). Substrate fertility was determined by the content of phosphorus (P), potassium (K), calcium and magnesium (Ca + Mg) for the effective cation exchange capacity (CEC), CTC actual (t), CEC at pH 7 (T), the sum of bases (SB), saturation by bases (V), and organic matter (OM). Chemical analyses were conducted at the Soil Chemistry Laboratory of the Faculdade de Agronomia e Medicina Veterinária (FAV) of Universidade de Brasília (UnB), following a previously described protocol [17].

Physical quality was evaluated by measuring volumetric humidity (θ), density (Ds), and total porosity (PT). To determine these variables, undisturbed samples were collected with a volumetric ring of known volume (9.25 cm^3^), transferred to aluminum cans, and weighed while still in the field to obtain the wet mass (mu). Afterwards, the samples were taken to the Laboratório de Tecnologia da Madeira da Fazenda Água Limpa (FAL) of Universidade de Brasília (UnB), where they were dried in a forced circulation oven at 105°C for 48 h to obtain the dry mass (ms). Using this information, θ and PT were determined using the following equations:

$$\theta =\frac{{(m}_{u}-m_{s})}{V_{ring}}$$ (2)

where θ = volumetric humidity (cm^3^ cm^-3^); mu = wet mass (g), ms = dry mass (g), and Vring = ring volume used to collect the undisturbed sample (cm^3^).

$$Ds=\frac{m_{s}}{V_{ring}}$$ (3)

where Ds = density (g cm^-3^) and Vring = ring volume used to collect the undisturbed sample (cm^3^).

$$PT=\left(1-\frac{Ds}{Dp}\right)$$ (4)

where PT = estimated total porosity (m^3^ m^-3^); Ds = soil density (Mg m^-3^); Dp = density of particles (Mg m^-3^), applying to dp the value of 2.65 g cm^-3^, the usual value used in the literature for mineral soils (4, 7).

### Data analysis

Initially, data were analyzed using a principal component analysis (PCA) to visualize relationships between physical/chemical quality and the four types of collection points. PCA is a multivariate statistical method that transforms an original set of correlated variables into a set of uncorrelated variables, called principal components (PC) while maintaining the maximum variability of the original set. The first PC explains a greater variability of the input dataset. The selection of the number of components used in the interpretation data with a common criterion indicates that components account for at least 70% of the variation [18].

Among the chemical variables evaluated, those that presented greater relevance in the discrimination of the evaluated factors were selected. Subsequently, comparisons of physical and chemical characteristics were performed at different collection points between the mined area and the adjacent Cerrado using means (Tukey) tests at 5% probability. It was first verified if the data had a normal error distribution and homogeneous variances, using the Jarque-Bera test with 10,000 permutations and Levene test, respectively. For data that met the normality and homogeneity of variances assumptions, differences between the levels of the analyzed factors were verified through an analysis of variance and Tukey’s test.

When the data did not meet the assumptions of normality and homogeneity of variances, statistically significant differences between the levels of the analyzed factors were determined using non-parametric tests to compare medians (Kruskal-Wallis and Mann-Whitney tests). Past v software, 2.17c, was used with a significance level of α <0.05.

## Results

### Growth and survival of planted seedlings

The 10 most abundant species in the restoration plantation presented a mean diameter of 30 cm from the soil (DB), ranging from 0.77 to 5.01 cm. HT ranged from 0.4 m to 1.8 m. *Tapirira guianensis* Aubl. (n = 63) and *Tapirira guianensis* Aubl. (n = 27) had the highest number of individuals in the sample and the highest mean HT values (Table 1). Among the most abundant species, seven were characterized as typical of| forest physiognomies, and three were characterized by savanna physiognomies (e.g., *Dipteryx alata* Vogel, *Eugenia dysenterica* Mart. Ex DC., and *Hancornia speciosa* Gómez).

**Table 1:** Dendrometric characteristics of the 10 most abundant species recorded in the gravel during the restoration process, 6 years post-planting.

Species	Family	N	DB (cm)	HT (m)	PHYT
*Cecropia pachystachya* Trécul	Urticaceae	27	3.43 ± 0.44	1.8 ± 0.7	F
*Tapirira guianensis* Aubl.	Anacardiaceae	63	2.76 ± 0.28	1.7 ± 0.4	F
*Plathymenia reticulata* Benth.	Fabaceae	10	5.01 ± 2.82	1.4 ± 1.1	F
*Hancornia speciosa* Gómez	Apocinaceae	9	3.64 ± 0.55	1.2 ± 0.9	S
*Inga laurina* (Sw.) Willd.	Fabaceae	25	4.01 ± 1.59	1.2 ± 0.5	F
*Copaifera langsdorffii* Desf.	Fabaceae	15	2.14 ± 0.38	1.0 ± 0.6	F
*Dipteryx alata* Vogel	Fabaceae	22	2.00 ± 0.39	1.0 ± 0.4	S
*Alibertia edulis* (Rich.) A. Rich. ex DC.	Rubiaceae	10	0.96 ± 0.67	0.7 ± 0.5	F
*Pouteria ramiflora* (Mart.) Radlk.	Sapotaceae	12	2.32 ± 0.67	0.7 ± 0.5	F
*Eugenia dysenterica* Mart. Ex DC.	Myrtaceae	17	0.77 ± 0.41	0.4 ± 0.2	S
Overall average			1.14 ± 0.25	1.27 ± 0.09	-

N = number of individuals; DB = diameter at collection height; HT = total height; PHYT = phytophysiognomy of preferential occurrence (F = forest; S = savanna)

The overall mortality rate of seedlings planted in the gravel was 62.5% in the sixth year after planting. However in the third year after planting, it was 22.84% during an evaluation of mortality rate only. The mortality rates of the two most abundant species in the samples, *T*. *guianensis* and *C*. *pachystachya*, were 28.4% and 29.2%, respectively (Table 1).

This demonstrates the importance of long-term monitoring of areas subjected to forest restoration, mainly in gravel areas, because soil degradation conditions strongly threaten the establishment of seedlings.

### Physical quality of the substrate and mined soil under the Cerrado

The textural characterization of the composite samples from the soil under native Cerrado and the gravel substrate indicated that these could be classified as clayey, with a clay content higher than 48%.

The first and second main components contained the physical variable, density (Ds). Volumetric humidity during the dry season (θdry) and volumetric humidity during the rainy season (θrainy) presented eigenvalues of 2.04 and 1.11, respectively, explaining 79.00% of the total variation. Ds was correlated with the first major component (0.98 correlation), whereas the second major component was correlated with θdry (0.76) and θrainy (0.69). This PCA did not use total porosity (PT) because of its dependence on Ds (calculated based on Ds values).

**Fig 2:**
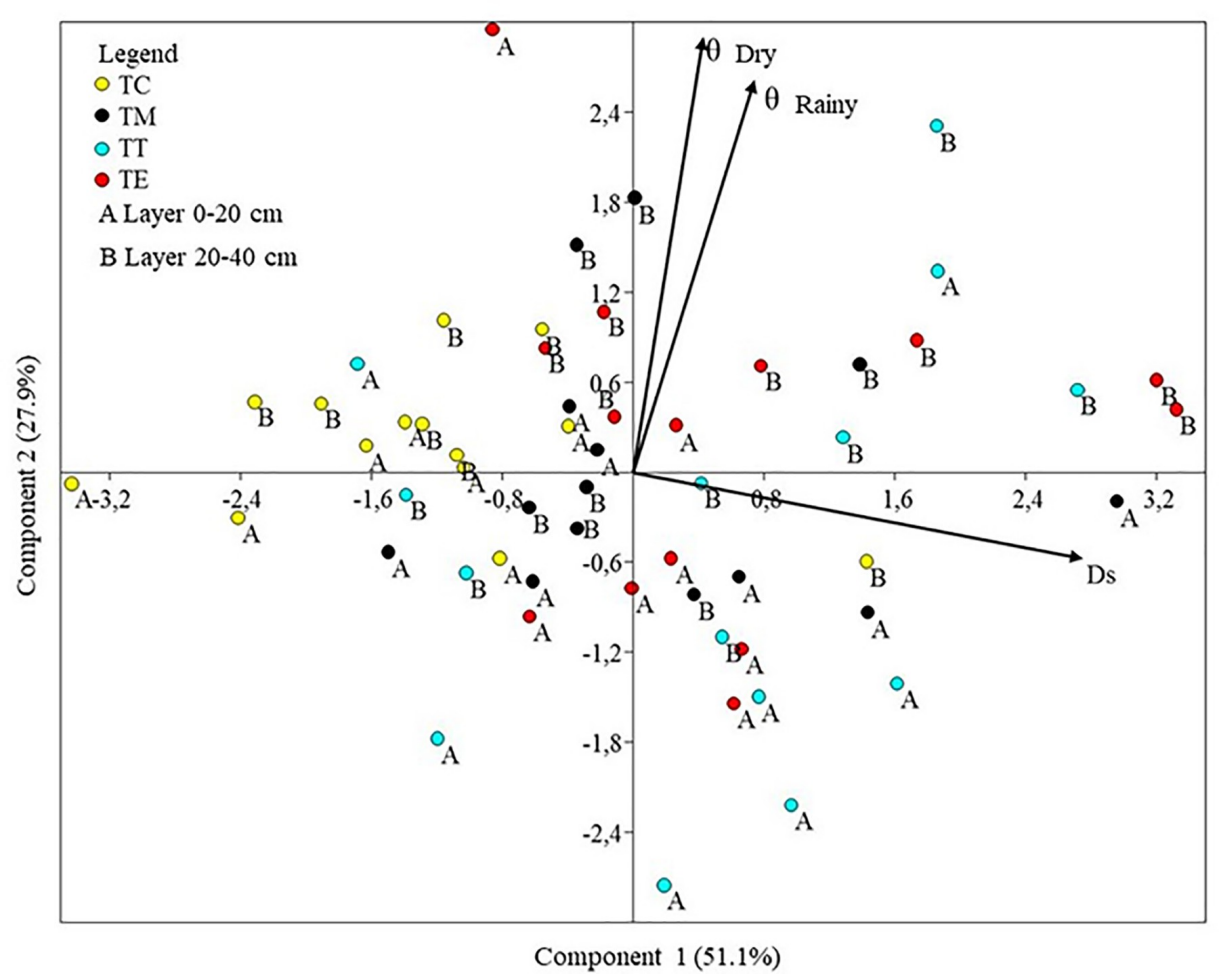
Principal component analysis (PCA) diagram of the physical variables. Ds = density; θdry = volumetric humidity in the dry season; θrainy = volumetric humidity in the rainy season; TC = samples taken from the soil under the Cerrado; TM = substrate samples taken from pits where seedlings did not survive; TT = substrate samples taken from *T*. *guianensis* pits; TE = substrate samples taken from *C*. *pachystachya* pits.

The diagram formed by the first two main components shows the grouping of soil samples taken from TC on the left side, indicating lower values of Ds. On the right side, the samples taken from the three types of gravel collection points (TM, TT, and TE) were concentrated without a clear separation between them. Most of the samples taken from the superficial layer (0–20 cm) of the gravel were concentrated in the lower part of the diagram, whereas samples taken from the subsurface layer (20–40 cm) were concentrated in the upper part of the diagram, close to the arrows indicating the humidity values (Fig 2).

The values observed for the means of Ds in the 0–20 cm layer varied between 0.93 (TC) and 1.19 g cm^-3^ (TT), whereas in the 20–40 cm layer, they varied between 1.00 (TC) and 1.24 g cm^-3^ (TE). Analysis of means showed that in the superficial layer, the average observed in the soil under TC was significantly lower than that observed in the pits where seedlings did not survive (TM) and in the *T*. *guianensis* pits (TT), which was not different from that observed in the *C*. *pachystachya* pits (TE) (F = 4.53; p = 0.01).

However, in the subsurface layer, the mean observed density for TC did not differ significantly from those observed for TM and TT, differing only from that observed for TE (F = 3.32; p = 0.03). When the density values in the layers were compared, no significant differences were observed between the surface (0–20 cm) and subsurface (20–40 cm) layers for any of the four types of points evaluated. The total porosity exhibited the same behavior as density (Table 2).

**Table 2:** Physical characteristics of the soil in the Cerrado and different types of gravel collection points for the substrate at two depths in Paracatu-MG.

Properties		TC	TM	TT	TE	TC	TM	TT	TE
		0–20 cm				20–40 cm			
Density	g cm^-3^	0.93^a^	1.17^b^	1.19^b^	1.14^ab^	1.00^a^	1.11^ab^	1.2^ab^	1.24^b^
Total Porosity	cm^3^ cm^-3^	0.65^a^	0.56^b^	0.55^b^	0.57^ab^	0.62^a^	0.58^ab^	0.55^ab^	0.53^b^
θ (dry season)	cm^3^ cm^-3^	0.11^aB^	0.1^aB^	0.09^aA^	0.12^aA^	0.12^aB^	0.11^aB^	0.12^aB^	0.13^aB^
θ (rainy season)		0.14^aA^	0.17^aA^	0.14^aA*^	0.13^aA*^	0.16^aA^	0.19^aA^	0.17^aA*^	0.2^aA*^

TC = samples taken from the soil under the Cerrado; TM = substrate samples taken from pits where seedlings did not survive; TT = substrate samples taken from *T*. *guianensis* pits; TE = substrate samples taken from pits of *C*. *pachystachya*. Means followed by different lowercase letters on the rows are significantly different according to the Tukey test, comparing the collection points; moisture averages followed by different capital letters in the columns are significantly different according to the paired t-test; moisture averages followed by an asterisk in the rows are significantly different according to the Tukey test, comparing the layers (0–20 × 20–40 cm).

There were no statistically significant differences in volumetric humidity (θ) when comparing the four types of collection points in both layers. However, when the humidity between the layers was compared, there were statistically significant differences in TT and TE, with higher values occurring in the subsurface layer (20–40 cm) during the rainy season (F = 11.25, p = 0.00 and F = 25.95; p = 0.00, respectively).

There were also significant differences for this variable when the seasons were compared. For the surface layer, the paired tests indicated higher θ values in the rainy season (October to March) for TC (t = -2.50; p = 0.04) and TM (t = -9.36; p = 0.00), whereas in the subsurface layer, the values of θ were higher in the rainy for all sampled points (t = -2.72; p = 0.03; t = -5.59; p = 0.00; t = -3.34; p = 0.01; t = -7.22; p = 0.00) (Table 2).

Therefore, the results of the tests of means of the physical variables corroborated the pattern evidenced by the PCA, in which the soil under TC tended to present density values slightly lower than those observed in the gravel substrate (TM, TT, and TE), where the subsurface layer (20–40 cm) tended to store larger volumes of water than the surface layers (notably during the rainy season).

### Chemical quality of the mined substrate and soil under the Cerrado

Similar to the interpretation proposed by Sousa and Lobato [19], the results of the chemical characteristic analysis indicated that the soil under TC and the substrate from the pits in the gravel (TM, TT, and TE) could be described as acidic (pH <5.1) and dystrophic (V <50%), with very high exchangeable aluminum (Al^3+^) content, with Al^3+^ (m) saturations consistently greater than 75%. The total CTC (T) ranged from adequate/high for TC and low for TM, TT, and TE (Table 3).

**Table 3:** Chemical characteristics of the soil under the Cerrado and different types of gravel substrate collection points at two depths and two stations, Paracatu-MG.

Treatment	pH	P	K	H+Al	Al^3+^	Ca+Mg	SB	t	T	OM	V	M
	(H_2_O)	-- mg dm^-3^ --		------------------- cmol_c_ dm^-3^ -----------------						dag kg^-1^	---- % ----	
Dry season												
0–20 cm depth												
TC	4.2	1.7	120.9	15.7	3.8	0.2	0.5	4.4	16.2	2.8	3.4	86.7
TM	4.8	1.8	67.7	5.5	3.1	0.3	0.5	3.6	6.2	0.8	8.3	86.4
TT	4.5	9.7	109.8	4.6	3.1	0.7	0.9	4.3	5.2	2.1	20.5	75.2
TE	4.5	3.8	70.5	3.5	3.3	0.7	0.8	4.1	4.4	1.7	22.1	77.1
20–40 cm depth												
TC	4.4	1.5	76.3	12.0	3.0	0.1	0.4	3.4	12.3	2.0	3.5	88.7
TM	4.8	1.3	36.4	5.3	3.1	0.4	0.5	3.8	5.8	0.6	8.1	84.1
TT	4.6	2.3	68.1	7.7	3.6	0.4	0.7	3.9	7.9	0.7	6.1	84.6
TE	4.8	1.9	36.4	6.3	3.7	0.3	0.4	4.3	7.5	0.6	6.1	89.3
Rainy season												
0–20 cm depth												
TC	3.7	4.9	110.4	17.1	6.0	0.1	0.4	6.4	17.5	2.3	3.8	93.1
TM	4.0	2.5	71.6	6.4	3.5	0.2	0.4	3.8	6.9	0.7	7.3	88.6
TT	3.9	10.3	108.6	6.9	3.3	0.3	0.6	4.1	7.4	1.8	11.7	81.4
TE	3.9	4.8	71.4	7.6	3.9	0.3	0.6	4.6	8.1	1.5	9.0	87.8
20–40 cm depth												
TC	3.9	2.9	83.0	11.9	4.4	0.1	0.3	4.7	12.2	1.8	4.2	93.2
TM	4.0	1.6	57.1	5.9	3.5	0.2	0.3	3.8	6.1	0.4	6.3	92.8
TT	4.2	2.3	65.6	6.2	3.1	0.2	0.3	3.4	6.5	0.5	7.9	91.5
TE	4.1	2.0	57.1	6.2	3.5	0.1	0.3	3.7	6.4	0.4	6.8	91.6

TC = samples taken from the soil under the Cerrado; TM = substrate samples taken from pits where seedlings did not survive; TT = substrate samples taken from *T*. *guianensis* pits; TE = substrate samples taken from *C*. *pachystachya* pits.

**Fig 3:**
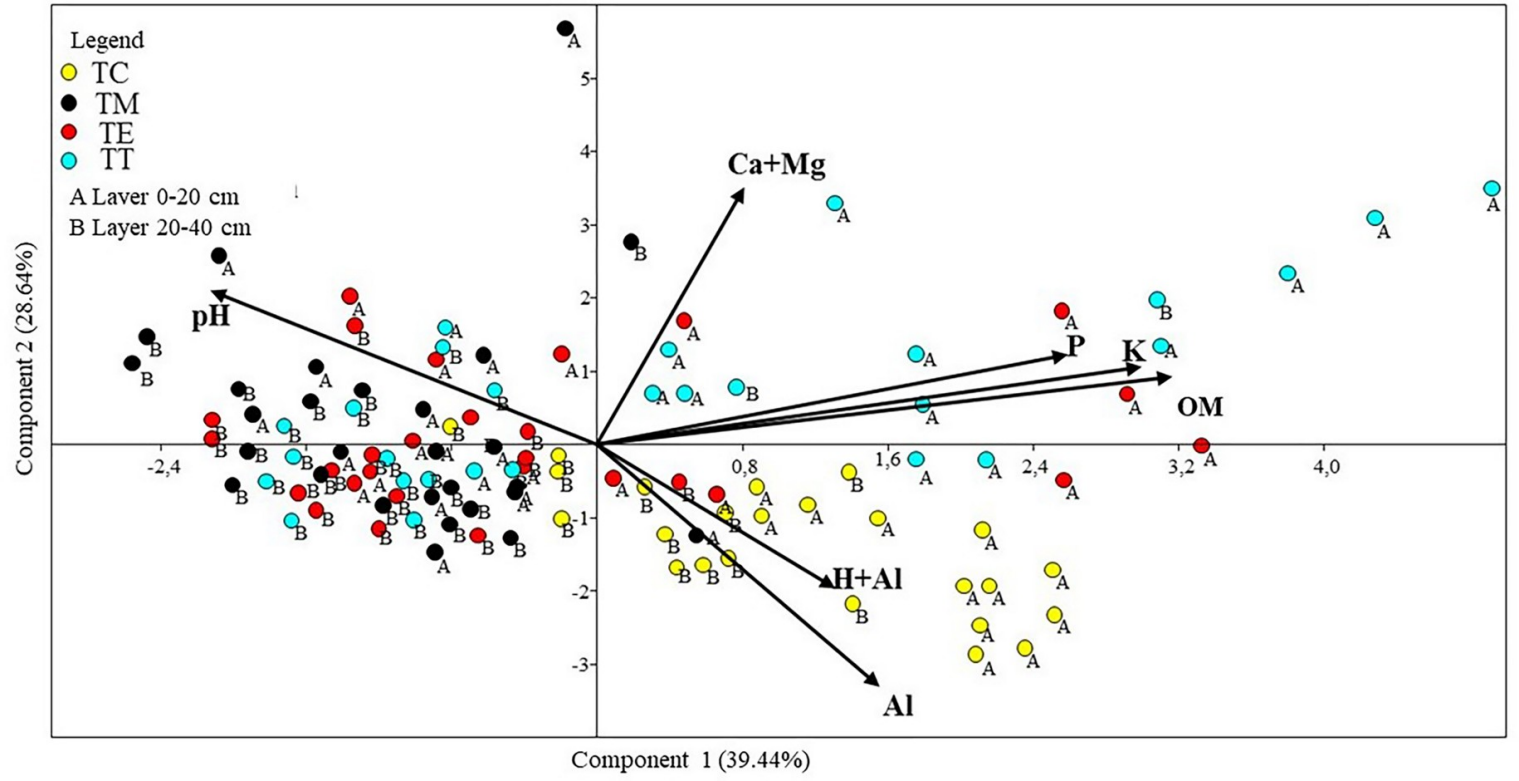
Chemical variables diagram. TC = samples taken from the soil under the Cerrado; TM = samples taken from the pit substrate where seedlings did not survive; TE = samples taken from the substrate of the *C*. *pachystachya* pits; TT = samples taken from the substrate of the *T*. *guianensis* pits.

The first three main PCA components, containing the chemical variables of the substrate, explained 79.52% of the total variation (Fig 3). The variables most correlated with the first component and their respective correlation values were OM (0.79), potassium (K) (0.74), and phosphorus (P) (0.63). The variables that were most correlated with the second main component and their respective correlation values were calcium and magnesium content (Ca + Mg) (0.81), exchangeable aluminum (Al^3+^) (-0.65), and potential acidity (H + Al) (-0.53). Regarding the third component, the most highly correlated variables were potential acidity (H + Al) (0.49), P (-0.46), and active acidity (pH) (0.40).

The diagram formed by the first two main components of the PCA (Fig 3) showed that samples taken in the surface layer (0–20 cm) of TT were concentrated in the first quadrant and associated with the arrows indicating P, K, and OM. In the fourth quadrant, samples taken from TC were grouped, in both the surface (0–20 cm) and subsurface depths (20–40 cm), and associated with the arrows indicating Al^3+^ and H + Al. This diagram also allows the visualization of correlations between chemical variables; arrows indicate P and K are close to the OM arrow and a negative correlation with pH.

## Discussion

### Growth and survival of planted seedlings

Regarding question (a), because of the distinct species characteristics and local conditions, the development observed by the seedlings planted in the gravel pits could be a reflection of insufficient cultural treatments in the pre- and post-planting stages, given the requirement for chemical and physical soil conditions to establish seedlings in gravel areas. This was verified by the significant difference in the density of the soil in the gravel when compared to the soil of the Cerrado *stricto sensu* and primarily by the higher contents of OM and potassium in an area of native vegetation.

In an assessment of the mortality rate performed in the third year after planting and documented by a local technical report by Centro de Referência em Conservação da Natureza e Recuperação de Áreas Degradadas, the mortality rate of 22.84% was lower than that found in the sixth year (62.5%). Possibly the seedlings would still be able to exploit the nutrients available through the fertilization of the pits. However, after this initial growth period, resources could have been exhausted, and the roots of many plants may have encountered adverse chemical/physical conditions because of the absence of horizons A and B in the soil, reflecting low growth and eventual mortality. Sanchez [6] reported that measures to correct acidity and fertilization conducted during the restoration process positively influenced the development of vegetation planted in mined areas.

The quality of seedlings is an important factor that could favor their establishment in the field, mainly in soils with physical restrictions. The success of restoration programs is directly associated with the morphophysiological quality of planted seedlings [20, 21]. *Tapirira guianensis*, a pioneer species that exhibited a greater abundance in the sampling of planted seedlings, has efficient mechanisms allowing tolerance to water deficits, such as the storage of water in its trunk and the maintenance of young leaves in its canopy during periods of drought, allowing better stomatal control [22].

*Cecropia pachystachya*, the second-most abundant species in the sampling of planted seedlings, is known to be a very efficient tree in establishing and colonizing open areas, such as clearings and degraded areas. It produces many seeds with a high percentage of germination in a few days [23]. It has been reported to be capable of stimulating succession because of the large number of animal species, such as birds and bats that use it for rest and food, enabling the transport of seeds of other species and pollination [24].

In contrast to the general mortality rate, which accounted for all the planted seedlings, *C*. *pachystachya* and *T*. *guianensis* seedlings had mortality rates below 30%. The performance of these species in the gravel has also been reported in other studies, such as those of Pilon & Durigan [25] and Salomão et al. [26], who highlighted their establishment and relatively good development in degraded environments. This confirms the need for the rapid recovery of substrates in mined areas.

### Physical quality of the mined substrate and soil under the Cerrado

Question (b) considered the physical properties of the substrate. In this case, the density values in the gravel pits were slightly higher compared to the Cerrado soil. Compaction is a frequent problem in mined areas, commonly caused by machine traffic on the bare substrate, sealing the porous space. However, the averages observed for the substrate density of the gravel pits varied from 1.11 to 1.24 g cm^-3^ and did not represent values limiting the growth of root systems because, for clayey substrates, the critical density limits vary between 1.4 and 1.8 Mg m^-3^ [27]. Additionally, the total porosity values, which ranged from 0.53 to 0.58 m^3^ m^-3^ in the gravel pits, were also close to those reported by Gomide et al. [28] and Giácomo et al. [29] in clayey soils under native vegetation in MG, where the average of this variable was approximately 0.55 m^3^ m^-3^.

From this, it can be inferred that the preparation of the gravel substrate, which consisted of revolving the substrate up to 60 cm in depth by subsoiling, provided decompression in the planting pits, reduced substrate density, increased total porosity, and promoted physical improvements during the pre-planting period, which were still seen in the sixth year after planting.

Regarding question (c), the analyses of the volumetric humidity averages (θ) in the collections conducted during the dry season indicated that all values of θ were less than or equal to 0.13 cm^3^ cm^-3^. In contrast, in the collections from the rainy season, the averages for this variable were between 0.14 and 0.20 cm^3^. This indicated that the values could be considered critical in the first season and could lead to the death of seedlings that are less tolerant to water deficits.

However, it is important to note that such critical limits have been proposed for agricultural systems, and their use as references for native Cerrado species should be done with caution because young plants of this genus are adapted to withstand seasonal water shortages in most surface soil layers [30]. For example, in the Cerrado Ralo (one of the Cerrado *stricto sensu* subdivisions), the existing vegetation may be naturally associated with soils with low water retention capacity [31], a situation that occurs in the remnant of the native Cerrado adjacent to the gravel area, and the θ contents observed in the samples taken from this remnant (TC) did not differ from those observed in the gravel area, suggesting that the humidity in this location was similar to that observed in areas where native plant communities were established.

In the Cerrado fragment, it was expected that higher levels of volumetric moisture would occur because of the greater coverage of the soil by trees and shrubs, which is associated with the interception of solar radiation, reducing evaporation of water from the soil. However, the θ values observed in the soil of this remnant indicated that it has a low water retention capacity in the evaluated layers compared to other Cerrado areas.

In general, the physical properties of the substrate of the gravel pits did not exhibit substantial variability based on the PCA plot and the comparison of means of density. The total porosity and volumetric humidity showed that the gravel substrate did not present significant differences at the three types of gravel collection points (TM, TT, and TE). Therefore, in the case of gravel pits, these variables did not indicate the most limiting properties for the establishment of seedlings in the study area.

### Chemical quality of the mined substrate and soil under the Cerrado

Chemical properties pertain to the answer for question “b”. In general, the substrates of the gravel pits have a chemical quality that can be considered limiting to the growth of seedlings, especially during the initial phase of growth in degraded soils, considering the low values of active acidity (pH <5.1) and phosphorus, negligible levels of calcium and magnesium (<0.7 cmol_c_ dm^-3^), and high levels of exchangeable aluminum (> 3.0 cmol_c_ dm^-3^) [19].

In the PCA, the opposite ordering of the TC in relation to gravel samples (TM, TE, and TT) could be related to planting fertilization, making it possible to increase the soil pH in the planting area. Planting fertilization made the pH of the gravel less acidic compared to the Cerrado area, which is a fundamental factor in differentiating these groups, which is driven by the higher levels of aluminum and OM in the Cerrado. Several authors have reported that planting fertilization, mainly by the phosphorus concentration, increases the soil pH [32, 33].

The OM content in the Cerrado is higher because of the cycle of senescence and decomposition of leaves, branches, and mortality of individuals over the years (citation needed). Under acidic conditions, the pH interacts with hydrogen (H^+^) and acts on the minerals releasing aluminum ions (Al^3+^) that are predominantly retained by the negative charges of the clay particles in the soil in equilibrium with the Al^3+^ in solution. Thus, the amount of Al^3+^ in the solution increases with the acidity.

Although significant differences were observed in the pH between the soil under TC (pH <4.5) and the gravel pits (TM, TT, and TE) in both seasons, considering the answer to “c,” these differences were more pronounced in the dry season. In this season, the active acidity in TC can be classified as very high (pH <4.5), whereas in TM, TT, and TE, they are classified as high (pH between 4.5 and 5.0) [34]. This property is classified as very high during the rainy season at all collection points (pH <4.5), being more limiting during this period. Higher active acidity (or lower pH values) during the rainy season may be caused by the hydrolysis of Al^3+^ in the soil solution [35], according to the following reaction:

$$Al^{3+}+2H_{2}O⇌Al{\left(OH\right)}^{2+}+H_{3}O^{+}$$

In addition to generating acidity by hydrolysis, excess Al^3+^ in soils is associated with toxicity to plants [36]. However, in the Cerrado biome, where naturally acidic soils often occur, there are native species tolerant or resistant to aluminum, which absorb large amounts of this element, whereas other species do not grow properly in the absence of Al^3+^ [37].

The species that grow and establish themselves in acidic soils are tolerant or resistant to aluminum, and their development and reproduction are not affected by the high levels of aluminum in the soil. Many common species of the Cerrado absorb and transport aluminum to leaves and accumulate it in different tissues, including leaves and seeds. In contrast, others do not survive in the absence of exchangeable aluminum, even though a specific role of Al in plant metabolism has not been established [38]. In mining areas, other factors can affect the survival and establishment of seedlings, such as water stress, damage from pests and diseases, and low microbial activity in the soil.

Although some species in this biome have developed mechanisms to adapt to excess Al^3+^ in the soil [39], planting in the gravel used several species of preferential occurrence in forest physiognomies, which are naturally associated with higher soil fertility and lower acidity. *Alibertia edulis* (Rich.) A. Rich. ex DC., for example, had relatively low averages for dendrometric variables, which could be explained by the effects of excess Al^3+^ present in the substrate. This author also reported that *T*. *guianensis* is more resistant to high levels of Al^3+^.

*Cecropia pachystachya* has also been shown to establish itself in places with limiting chemical conditions. Pinheiro et al. [40] assessed a mined area for sand extraction that had high activity (pH = 3.80) and potential acidity (H+Al = 9.5 cmol_c_ dm^-3^) and noted that the species was among the few trees initiating a successional process.

The comparisons of the P and OM contents at different collection points showed important soil-plant relationships. The relationship between TT and adequate phosphorus levels (P> 8.1 mg dm^-3^) and OM close to the soil under the Cerrado suggests that there was greater availability of nutrients in the *T*. *guianensis* pits. However, *C*. *pachystachya* proved to be a highly plastic species in relation to the substrate chemistry, managing to settle and grow in conditions similar to those that occur in pits where other plants did not survive.

The important role of OM and P as indicators of the substrate quality of mined areas has been highlighted in other studies evaluating areas in the restoration process. Almeida & Sánchez [6], studying gravel areas, showed that the critical limits for these variables were 1.4 dag kg^-1^ and 2.58 mg dm^-3^, respectively. The relevance of OM as a soil conditioner is well recognized in the literature and is associated with improved CTC, reduced Al^3+^ toxicity through complexation, reduced H^+^ activity with a consequent reduction in acidity, and reduced P adsorption by highly weathered soils [41]. Thus, adding OM to ecological *e*.*g*., agricultural residues, such as crop straw, forest harvest residues, litter transposition.

P levels below 3.0 mg/dm^3^ suggest that removing the surface soil horizons led to the loss of this nutrient. However, low levels of this nutrient have also been found in TC soil. Novais et al. [42] stated that in soils in tropical regions, where weathering is intense, it is common to find high amounts of Fe and Al oxyhydroxides and a pronounced clay fraction. These characteristics cause problems in P adsorption, resulting in non-labile forms of the element in the soil. This phenomenon can also intensify at low pH values because of the presence of positive charges that favor adsorption.

The results showed a close correlation between OM and P, as shown in the PCA diagram (Fig 3). Thus, OM deficiency is a factor that requires significant efforts during a project to restore degraded substrates, as several studies have shown [43]. The removal of vegetation and surface and subsurface layers of the soil causes the loss of OM and mineral nutrients; however, Al^3+^ reduces the pH, makes the environment acidic and dystrophic, and limits the establishment of vegetation as observed in the study area. Regarding the K content, it was noted that the removal of superficial horizons in the gravel caused a reduction in the availability of this nutrient compared with the patterns observed in TC.

The chemical quality of the substrate and the compromised physical structure of soil resulted in high mortality in the different species. Thus, to avoid the death of seedlings caused by the low chemical quality of the substrate in degraded areas, it is recommended to correct acidity and fertility and promote the maintenance of higher levels of nutrients through additional cover fertilization [44, 45]. In this context, Pinheiro et al. [40] argued that the use of correctives and agricultural fertilizers only when planting seedlings might not be efficient in the long-term in mined areas because of the temporary availability of large amounts of nutrients. Additionally, it inhibits the association between plants and microorganisms. Although some economic and technical limitations can influence the evolution of the restoration process. Thus, it’s important to consider the particularity of kind situation.

Regarding the SB components, Ca + Mg contents were classified as very low at all collection points, at both depths, and during both seasons [19, 46]. Therefore, correcting the acidity of the substrate by applying lime to the planting pits would enable better growth of the planted seedlings with the use of more demanding native species seedlings for plantings.

## Conclusions

The following conclusions summarize our results:

- The low growth in height and diameter and the high mortality rates of most planted seedlings revealed that six years after planting, the establishment was significantly impaired by the conditions of the degraded area.
- In the soil under the Cerrado, physical soil quality similar to the substrate of the gravel pits was observed, indicating that the investigated properties (density, total porosity, and volumetric humidity) analyzed in isolation were not limiting in the gravel pits.
- The soil under the Cerrado had better OM levels, whereas in the gravel pits where the seedlings died, the lowest levels were observed, indicating the important role of OM as a soil conditioner and source of nutrients for plants.
- *T*. *guianensis* seedlings were associated with higher phosphorus and OM levels in the substrate, whereas *C*. *pachystachya* proved to be a highly plastic species in relation to substrate chemistry, surviving in conditions that other plants were unable to withstand.
- The presence of high levels of exchangeable aluminum that undergoes hydrolysis during the dry season makes soil acidity a more limiting property to the growth of planted seedlings, especially those that do not have mechanisms for tolerance to Al^3+^ toxicity.
